# Willingness to Pay for Dog Rabies Vaccine and Registration in Ilocos Norte, Philippines (2012)

**DOI:** 10.1371/journal.pntd.0004486

**Published:** 2016-03-21

**Authors:** Meseret G. Birhane, Mary Elizabeth G. Miranda, Jessie L. Dyer, Jesse D. Blanton, Sergio Recuenco

**Affiliations:** 1 Poxvirus and Rabies Branch, Division of High-Consequence Pathogens and Pathology, National Center for Emerging and Zoonotic Infectious Diseases, Centers for Disease Control and Prevention, Atlanta, Georgia, United States of America; 2 Global Alliance for Rabies Control, Manhattan, Kansas, United States of America; Swiss Tropical and Public Health Institute, SWITZERLAND

## Abstract

**Background:**

The Philippines is one of the developing countries highly affected by rabies. Dog vaccination campaigns implemented through collaborative effort between the government and NGOs have played an important role in successfully reducing the burden of disease within the country. Nevertheless, rabies vaccination of the domestic animal population requires continuous commitment not only from governments and NGOs, but also from local communities that are directly affected by such efforts. To create such long-term sustained programs, the introduction of affordable dog vaccination and registration fees is essential and has been shown to be an important strategy in Bohol, Philippines. The aim of this study, therefore, was to estimate the average amount of money that individuals were willing to pay for dog vaccination and registration in Ilocos Norte, Philippines. This study also investigated some of the determinants of individuals’ willingness to pay (WTP).

**Methods:**

A cross-sectional questionnaire was administered to 300 households in 17 municipalities (out of a total of 21) selected through a multi-stage cluster survey technique. At the time of the survey, Ilocos Norte had a population of approximately 568,017 and was predominantly rural. The Contingent Valuation Method was used to elicit WTP for dog rabies vaccination and registration. A ‘bidding game’ elicitation strategy that aims to find the maximum amount of money individuals were willing to pay was also employed. Data were collected using paper-based questionnaires. Linear regression was used to examine factors influencing participants’ WTP for dog rabies vaccination and registration.

**Key Results:**

On average, Ilocos Norte residents were willing to pay 69.65 Philippine Pesos (PHP) (equivalent to 1.67 USD in 2012) for dog vaccination and 29.13PHP (0.70 USD) for dog registration. Eighty-six per cent of respondents were willing to pay the stated amount to vaccinate each of their dogs, annually. This study also found that WTP was influenced by demographic and knowledge factors. Among these, we found that age, income, participants’ willingness to commit to pay each year, municipality of residency, knowledge of the signs of rabies in dogs, and number of dogs owed significantly predicted WTP.

## Author Summary

Rabies is one of the most fatal viral diseases mostly transmitted through a bite of an infected mammal. In many parts of Africa and Asia, rabid dogs are the main transmitters of the disease to humans. In the Philippines, government enforced dog registration laws and government/NGO sponsored mass dog vaccination campaigns have so far been the cornerstone in successfully reducing the burden of disease in parts of the country. To further enhance the continuation of such programs, however, an introduction of affordable dog vaccination and registration fees to the public is vital and have shown to be an important strategy in other parts of the country. Our main objective here was to systematically assess how much money individuals were willing to pay for dog vaccination and registration in Ilocos Norte, Philippines. We also assessed whether the amount individuals were willing to pay was influenced by their demographic and knowledge characteristics. We conducted a cross-sectional study using a combination of cluster sampling, random sampling, and convenience sampling methods to identify study participants. We employed a specific elicitation strategy (bidding game) to elicit how much individual were willing to pay for such services. Our results indicate that Ilocos Norte residents, on average, were willing to pay 69.65 PHP (1.67 USD) for dog vaccination and 29.13 PHP (0.70 USD) for dog registration. The majority (86%) of respondents were willing to pay the stated amount to vaccinate each of their dogs, annually. We also found that willingness to pay was influences by age, income, municipality of residency, people’s willingness to commit to pay each year, number of dogs owned, and knowledge regarding rabies signs in dogs. This findings give policy makers some indication of how much people were willing to contribute financially towards dog vaccination and registration in particular and towards rabies elimination from Ilocos Norte, in general. Socio-economic and demographic factors, however, may need to be considered prior to the introduction of such fees to the public.

## Introduction

Rabies is an acute, viral zoonosis globally responsible for more than 59,000 deaths annually [1]. Once clinical symptoms develop, the disease has one of the highest case fatality ratios of any infectious disease. The majority of all human deaths from rabies occur in Africa (36.4%) and Asia (59.6%) where canine rabies virus variants are predominant [1]. Transmission of rabies virus from dogs accounts for more than 90% of human cases [2].

Another important aspect of the impact of rabies is its economic burden that arise from disease prevention efforts and mortality cost in humans, livestock, and other domestic animals. In Asia alone, the human mortality cost of rabies is estimated to be approximately 67.87 billion US dollars annually [3]. Although dog rabies vaccination is considered as the most cost-effective solution to prevent rabies deaths in humans, the vaccination coverage of dogs in some Asian countries remain as low as 33% which is well below the suggested necessary coverage limit of 70% to obtain herd immunity [4–6].

The Philippines is one of the developing countries highly affected by rabies where, annually, an estimated 200–300 human deaths are attributed to rabies [7]. Rabies prevention and control policies and dog vaccination campaigns are currently the cornerstone of rabies elimination strategy in this country. The “Anti-rabies Act of 2007” with the objective of eliminating rabies throughout the Philippines by 2020 was enacted by the government of Philippines in 2007 [8]. Dog vaccination campaigns have so far been shown to be an effective rabies prevention and control strategy in reducing the burden of disease in parts the country [9]. In particular, the Bohol Rabies Prevention and Elimination Program (BRPEP), with the support of the local government and international Non-Government Organizations (NGOs), was able to considerably reduce human rabies in the province of Bohol. This was made possible through effectively utilizing social awareness campaign, dog population control measures, dog registration and mass dog vaccination campaigns, in addition to improved dog bite management and veterinary quarantine services [10]. Similar models of rabies elimination have also been initiated in other provinces of the Philippines. The Communities Against Rabies Exposure (CARE) Project, with the aim of creating another rabies free zone using similar rabies elimination strategies implemented in Bohol, was launched in Sorsogon Peninsula and Ilocos Norte in 2012 [11]. In 2014, with yet another support from the local government and international NGOs, this program have successfully incorporated rabies prevention messages into the elementary school curriculum and vaccinated 35 per cent of some 76,000 dog population in the province of Ilocos Norte [12, 13]. Through this program, the province of Ilocos Norte has been rabies-free since 2013 [13]. Nevertheless, long-term rabies elimination from an area requires recurrent implementation of mass dog vaccination campaigns that can help maintain the herd immunity in a given population. To achieve this, a multi-year commitment is required not only from governments and NGOs, but also from the local community that directly benefit from such efforts. An introduction of affordable dog vaccination and registration fees to the public is therefore essential and has been shown to be an important strategy in Bohol [10].

## Methods

### Study site and population

**Fig 1:**
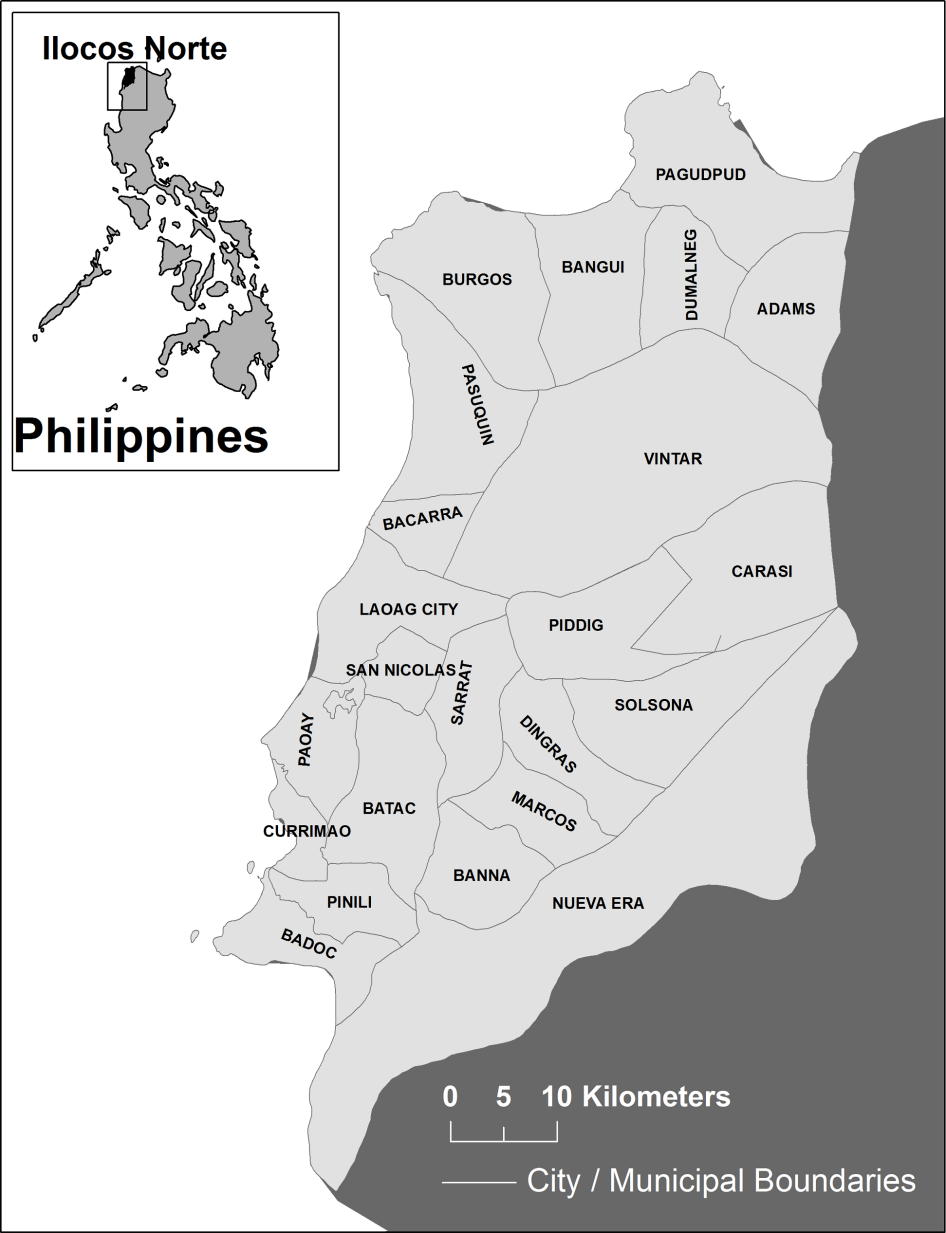
Map of the province of Ilocos Norte, Philippines.

A cross-sectional study was conducted in Ilocos Norte located in the northernmost province on the western side of Luzon, Philippines (Fig 1). Ilocos Norte had an estimated population of 568,017 in 2010 and is predominantly rural (2010 census data) [14]. The annual average family income is 204,000 Philippine Pesos (PHP) (4,334 USD) and the annual average family expenditure is 159,000 PHP (3,378 USD) (2012 census data) [15]. The survey was conducted over a period of two weeks in August 2012 during the peak of the rainy season.

To identify participants, combinations of random sampling, cluster sampling, and convenience sampling methods were employed in three sequential steps. Cluster sampling with probability proportionate to size (PPS) was used to identify villages (locally known as barangays). A random sampling method was used to identify roads (locally known as puroks) and the first participating household in the cluster. Convenience sampling was subsequently used to identify the remaining nine households as well as actual participants within households. This methodology was particularly employed because complete randomization of households was not feasible due to the vastness of the study area and population.

The identification of the village was carried out by creating a cumulative list of community populations and selecting a systematic sample from a random start. A list of all the villages in Ilocos Norte and their corresponding population size was first obtained from a census data and then the villages were arranged in an alphabetical order. A sampling interval (SI) was calculated by taking the ratio of the cumulative population of Ilocos Norte to the total number of clusters (thirty). A random start number was then generated to obtain the first participating village. This village had a population closest to but not greater than the random number. Subsequently, the SI was added to the previously identified random number in order to obtain the second village. The third village was again identified by adding the SI to the preceding random number. This process was repeated again and again until the 30^th^ village (cluster# 30) was identified. Through this process, a total of 30 villages in 17 municipalities (out of a total of 21 municipalities) were identified and included.

A road in a particular village was randomly selected based on a list obtained from the respective village officials. The same list also included households within the selected roads and served as the sampling frame for randomly selecting the first participating household around which a cluster of households was further identified. A total of 10 households in each road were included in the survey. Participants were then selected by convenience sampling according to the inclusion criteria (respondents must be equal to or greater than 18 years of age). Only the head of the household or the next representative household member were interviewed. These participants were purposively selected to be interviewed because it was believed that they play an important role in the decision making process of the household.

### Sample size determination

To determine the appropriate sample size for this survey, an estimated population proportion of 20%, with ±5% confidence interval and 95% and coefficient was used. In addition, a cluster size of 10 with rate of homogeneity at 0.2 was employed. Based on a priori evidence, we anticipated a design effect of 1.18 [9].

### Human research considerations

Human subjects’ clearance for this study was obtained from the Centers for Disease Control and Prevention (CDC) Human Research Protection Office in Atlanta, United States, under CDC protocol #6337 as well as the Mariano Marcos Memorial Hospital and Medical Center Ethics Review Committee in Batac City, Ilocos Norte under the protocol number 2012-07-014, and was determined exempt from full Institutional Review Board (IRB) review.

Written informed consent was obtained from all participant prior to commencement of the study. If the participant was unable to read and write, the consent form was read to the participant and a thumbprint was obtained in place of a signature. The age of consent in the Philippines is 18 years. Therefore only participants 18 years old and over were allowed to participate in this study.

### Survey instruments

A paper based questionnaire was administered in Ilocano, the local language of the majority of Ilocos Norte’s population (2002 census data). Interviews were conducted by representatives from CDC Atlanta, in collaboration with the Provincial Veterinary Office in Ilocos Norte. A total of 23 interviewers participated in conducting the survey. Interviewers were pre-trained on the survey methodology used and the questionnaire was pre-tested in the field in order to evaluate its workability, and appropriate modifications were made. Responses obtained from the interview were subsequently translated into English for analysis.

The questionnaire covered four major categories relevant to this analysis. The first category consisted of questions regarding household dog(s). The second category of questions, the WTP section, included an introductory statement explaining the purpose and importance of dog vaccination and registration campaigns and collected information on WTP for dog rabies vaccination and registration accordingly. In this section, the contingent valuation method (CVM) was used to elicit WTP for dog rabies vaccination and registration. In the context of health care, the CVM is a survey-based, hypothetical and direct approach to elicit monetary value to improve goods and services. Contingent valuation questions are used to estimate the willingness to pay distribution of consumers towards specific goods/services. It is a stated preference model that can measure the value consumers place on certain aspects of health care services[16, 17].

**Fig 2:**
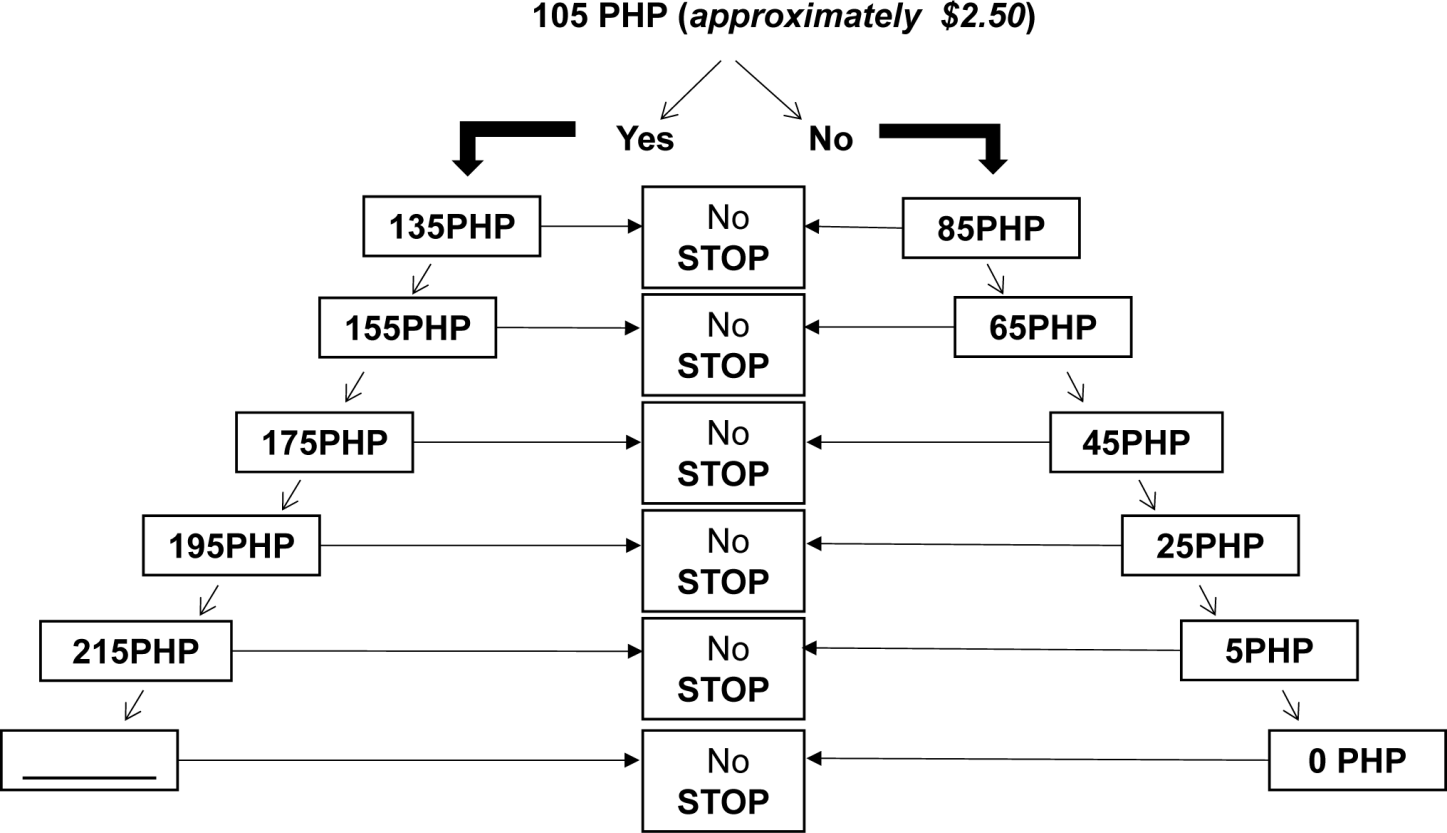
Bidding game algorithm. An example of the bidding game algorithm with a randomly generated starting point of 105 PHP (2.50 USD) used to obtain participants’ maximum WTP for vaccination price.

In this survey, a particular elicitation method, *a bidding game* [18] with a series of yes/no questions that aim to find the maximum WTP was employed. This method was chosen because it was expected to have criterion validity (which here refers to whether the instrument of measurement adequately represents the object of measurement) in the setting of Ilocos Norte where there was an established culture of price negotiation for most goods [19]. Furthermore, empirical studies have found this method to be very reliable [20, 21]. During the process of the bidding game, the participants were first offered an initial maximum WTP price. If the respondent accepted the initial price, a series of higher prices were offered until the respondent rejected the price. Alternatively, if the respondent refused the initially offered price, then the prices were repeatedly decreased until the respondent accepted or reached zero (Fig 2). For a more accurate estimation of the maximum WTP, the bid was presented in Philippines Pesos (PHP). During the bidding process, a uniform distribution of 12 bid levels was presented for the WTP for vaccination section, and 10 bid levels were presented for the WTP for registration section. Each increasing and decreasing level had a difference of 20PHP (~ 0.50 US dollars) [22]. To minimize potential starting point bias, when the offered start bid influences the direction of the WTP, the initial biding value offered was selected randomly using random number generation at the interview site. The interviewers were pre-trained in applying the randomization processes as well as in conducting the bidding game.

The third and the fourth category comprised of sets of questions that aimed to explore determinants of WTP. Specifically, these sections looked at the demographic characteristics of survey respondents (gender, age, employment status, household income, and dog ownership status) and explored participants’ awareness of rabies transmission, exposure, and outcome.

### Data analysis

Paper surveys were digitized and a database was built in Microsoft Access. Accuracy of the data was subsequently checked to minimize data entry error. Descriptive statistics of the demographics of the study population were calculated.

Linear regression was used to examine factors influencing participants’ WTP for dog rabies vaccination and registration. Linear regression diagnostics were first performed to check how well the data met the assumptions of the linear regression. Normality of the data was tested using graphical methods of residuals versus fitted (predicted) plot. Cook-Weisberg test was used for detecting heteroscedasticity [23, 24]. Since the data violated the linear regression assumptions of both normality and heteroskedasticity, an attempt to attain the validity of that assumption was made using power transformation and robust standard errors, respectively. The appropriate power transformation of the response variable was determined using Tukey’s ladder of power [25]. Accordingly, a power transformation of 0.5 was used for the approximation of the residuals of the response variable to normality. Kernel density plots were then generated against each continuous predictor variables to assess if the predictor variable satisfies the linearity assumption [26]. Predictor variables that violated this assumption were further transformed using the appropriate power transformation. The analysis also accounted for the sampling methodology used (cluster sampling) and standard errors were adjusted during the model building. Accordingly, cluster robust standard errors that put into consideration the clustering effect were used during the analysis.

All predictor variables that remained significantly associated (P≤0.1) in the univariate model were retained (variables tested are presented in Tables 4 and 5). Multicollinearity among predictor variables was assessed for using Variance Inflation Factor (VIF). As a rule of thumb, a tolerance value (1/VIF) of lower than 0.1 (or VIF greater than 10) was used as a cutoff point to check if some level of multicollinearity existed. All of the VIF scores were less than 1.5 and therefore no signs of multicollinearity was observed between predictor variables. The predictor variables were then fitted into a full model and further reduced through backward selection at a 5% significance level to obtain the final model. Coefficients and direction of the linear association between variables were determined from the final model and back transformed to the original scale. Mean and range values of WTP for vaccination and registration were calculated using the transformed data. The sample mean of the transformed data together with its 95% confidence interval was then back transformed to obtain the population median and its corresponding 95% confidence interval of the original data [27]. Statistical analyses were performed using STATA version 13 (Statacorp, Texas, USA).

## Results

### Demographics

A total of 300 respondents were included in the study (Table 1). The majority of the respondents were female (65%) and the mean age was 48 (SD 16). Eighty six percent of the respondents reported an annual income of less than 120,000PHP (2,876USD). In addition, the majority of respondents owned dogs (n = 64%). The average number of dogs owned by a household was 1.3 (SD 1.6) and the dog to human ratio was 1:3.5. Therefore, the estimated owned dog population of Ilocos Norte is expected to be 162,290 utilizing the predicted dog to human ratio.

**Table 1:** Demographic characteristic of survey respondents and their households, Ilocos Norte, Philippines 2012.

Characteristics	N	n (%)	Mean (SD, range)
**Age**	298		48 (16, 20–90)
**Gender**	299		
Male		106 (35.45)	
Female		193 (64.55)	
**Employment Status**	297		
Employed		244 (82.15)	
Unemployed		53 (17.85)	
**Household annual income (PHP)**	292		
120,000 and below		251 (85.96)	
120,001–180,000		28 (9.59)	
180,001–240,001		5 (1.71)	
240,001–300,000		3 (1.03)	
300,001–360,000		2 (0.68)	
360,001–420,000		1 (0.34)	
420,001 and above		2 (0.68)	
**Dog ownership status**	300		
Own Dog/s		192 (64)	
Does not own dog/s		108 (36)	
**Number of persons/household**	298		4.6 (2.24, 1–15)
**Number of dogs/household**	300		1.3 (1.6, 0–9)
**Number of samples per Municipality**			
Bacarra		20(6.67)	
Badoc		20(6.67)	
Banna		10(3.33)	
Batac		30(10.00)	
Burgos		10(3.33)	
Dingras		20(6.67)	
Laoag City		60(20.00)	
Marcos		10(3.33)	
Pagudpud		10(3.33)	
Paoay		20(6.67)	
Pasuquin		10(3.33)	
Piddig		10(3.33)	
Pinili		10(3.33)	
San Nicolas		20(6.67)	
Sarrat		10(3.33)	
Solsona		20(6.67)	
Vintar		10(3.33)	

Forty three percent of the dog owners stated that they have vaccinated at least one of their dogs once within the past two years. Out of these, 8% stated that they have previously paid for their dog(s) to be vaccinated.

In regards to rabies awareness, almost all (99%) of the respondents have heard of rabies and the majority (69%) knew how to recognize the clinical signs of rabies in dogs. Signs and outcomes of rabies in humans were adequately identified by 50% and 63% of the respondents, respectively (Table 2). This results varied by gender. Females were significantly more aware of the signs and outcome of rabies in humans than males. However, only 46% of respondents knew how rabies was transmitted to humans. There were no significant discrepancies in awareness of rabies between those who own dog(s) and those who did not (Table 2).

**Table 2:** Knowledge of human and canine rabies among survey participants by gender and dog ownership status, Ilocos Norte, Philippines 2012.

			Gender			Dog ownership status		
Rabies knowledge assessment questions	N	Overall n (%)	Male n (%)	Female n (%)	Χ^2^	Does not own a dog(s) n (%)	Own dog(s) n (%)	Χ^2^
**Have you ever heard of the disease called rabies?**	299				0.615			0.662
No		4 (1.34)	2 (1.90)	2 (1.04)		2 (1.85)	2 (1.05)	
Yes		295 (98.66)	103 (98.10)	191 (98.96)		106 (98.15)	189 (98.95)	
**What are the signs of rabies in dogs?**	296				0.076			0.464
Don’t know		81 (27.36)	33 (31.73)	47 (24.61)		33 (31.13)	48 (25.26)	
Limited recognitionᵃ		12 (4.05)	1 (0.96)	11 (5.76)		5 (4.72)	7 (3.68)	
Adequate recognitionᵇ		203 (68.58)	70 (67.31)	133 (69.63)		68 (64.15)	135 (71.05)	
**How does a person get rabies?**	294				0.386			0.364
Don’t know		157 (53.40)	60 (57.69)	97 (51.05)		51 (48.57)	106 (56.02)	
Limited recognitionᵓ		2 (0.68)	0 (0.00)	2 (1.05)		1 (0.95)	1 (0.53)	
Adequate recognitionᵈ		135 (45.92)	44 (42.31)	91 (47.89)		53 (50.48)	82 (43.31)	
**What are the signs of rabies in person?**	291				**0.018**			1.00
Don’t know		138 (47.42)	59 (57.84)	79 (41.80)		50 (47.62)	88 (47.31)	
Limited recognitionᵃ		7 (2.41)	3 (2.94)	4 (2.12)		2 (1.90)	5 (2.69)	
Adequate recognitionᵇ		146 (50.17)	40 (42.31)	106 (56.08)		53 (50.48)	93 (50.00)	
**Have you ever known anyone who had rabies?**	298				0.685			0.907
Yes		79 (26.51)	25 (23.81)	54 (27.98)		29 (26.85)	50 (26.51)	
No		212 (71.14)	77 (77.33)	135 (69.95)		76 (70.37)	136 (71.14)	
Don’t know		7 (2.35)	3 (2.86)	4 (2.07)		3 (2.78)	4 (2.35)	
**What happens to most people who become ill with rabies?**	293				**0.006**			0.172
Don’t know		74 (25.26)	33 (31.73)	41 (21.69)		20 (19.05)	54 (28.72)	
Limited recognitionᵐ		35 (11.95)	18 (17.31)	17 (8.99)		13 (12.38)	22 (11.70)	
Adequate recognitionᵑ		184 (62.80)	53 (50.96)	131 (69.31)		72 (68.57)	112 (59.57)	

ᵃThis category comprises of individuals who stated symptoms that are not specific to rabies.

ᵇThese category comprises of individuals who stated at least one rabies related symptom.

ᵓThis category comprises of individuals who stated exposures that are not specific to rabies.

ᵈThese category comprises of individuals who stated at least one rabies related exposure.

ᵐThis category comprises of individuals who stated outcomes that are not specific to clinical rabies.

ᵑThese category comprises of individuals who identified any clinical manifestations of rabies or stated “death” as the clinical outcome of rabies in humans.

### Willingness to pay for dog vaccination and registration

Bidding games were completed by almost all (298 of the 300) respondents to elicit their maximum WTP for dog vaccination and registration (Table 3). Two people withdrew from the interview due to the inconvenience associated with the length of the interview. Eighty eight percent of the WTP for dog vaccination and 86% of WTP for dog registration responses were above zero. The odds of participants stating they were willing to pay for dog registration was almost 30 times (CI; 13 to 70) higher among those who were willing to pay for dog vaccination compared to those who were not willing to pay for vaccination.

**Table 3:** Willingness to pay for rabies vaccination and registration for dogs in PHP^ᵖ^ among 298 survey participants, Ilocos Norte, Philippines, August 2012.

WTP for:	N(%)	Population median [Range]ᵗ	95% CIᵘ	Population median [Range]ᵛ	95% CI
Vaccination	298 (99.33)	69.65 [0–500]	[57.70; 82.73]	1.67 [0–11.98]	[1.38;1.98]
Registration	298 (99.33)	29.13 [0–300]	[26.01; 32.43]	0.70 [0–7.19]	[0.62;0.78]

^ᵖ^PHP = Philippines Peso

ᵗEstimates in Philippines Pesos

ᵘCI = Confidence Interval

ᵛEstimates in United States Dollars (USD), exchange rate at time of survey PHP41.72 = 1USD (www.fms.treas.gov)

**Fig 3:**
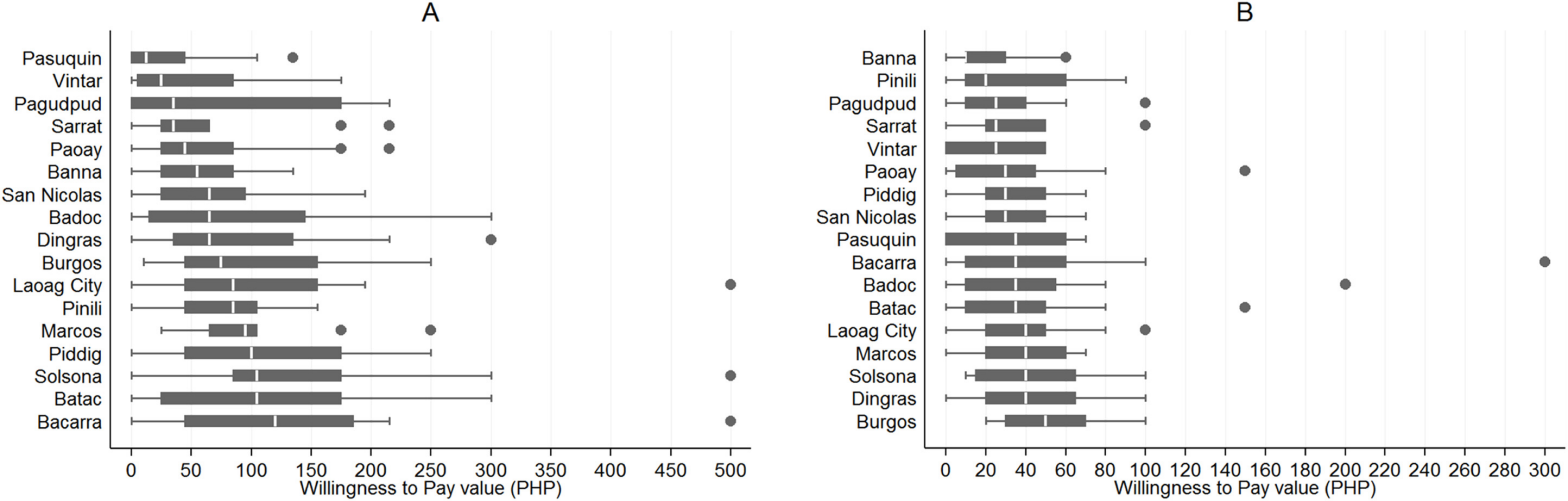
**A. Distribution of the maximum WTP for dog vaccination within the 17 municipalities, Ilocos Norte, Philippines.** B. Distribution of the maximum WTP for dog registration within the 17 municipalities, Ilocos Norte, Philippines.

The population medians for the WTP for dog vaccination and registration were estimated to be 69.65 PHP (1.67 USD) and 29.13 PHP (0.70 USD), respectively (Table 3). Looking at the distribution of the WTP medians across the selected municipalities in Ilocos Norte, the lowest median WTP for vaccination value was observed in the municipality of Pasuquin while the highest was in the municipality of Bacarra. For dog registration, the lowest and the highest medians were observed in the municipality of Banna and Burgos, respectively (Fig 3A and 3B).

A good majority of those who owned dog(s) were willing to pay the stated amount for dog vaccination and/or registration. Only 10% and 14% of dog owners had a stated maximum WTP of zero PHP for vaccination and for registration, respectively.

In general, the majority of respondents (86%) indicated they were willing to pay the stated amount to vaccinate each of their dogs annually, while the remaining proportion were either not willing to accept this commitment (12%) or didn’t know (2%) if they wanted to commit. Eighty-six percent of dog owners were willing to commit. The same percentage of those who did not own dog/s where also willing to commit to pay for each of their dogs, annually.

**Fig 4:**
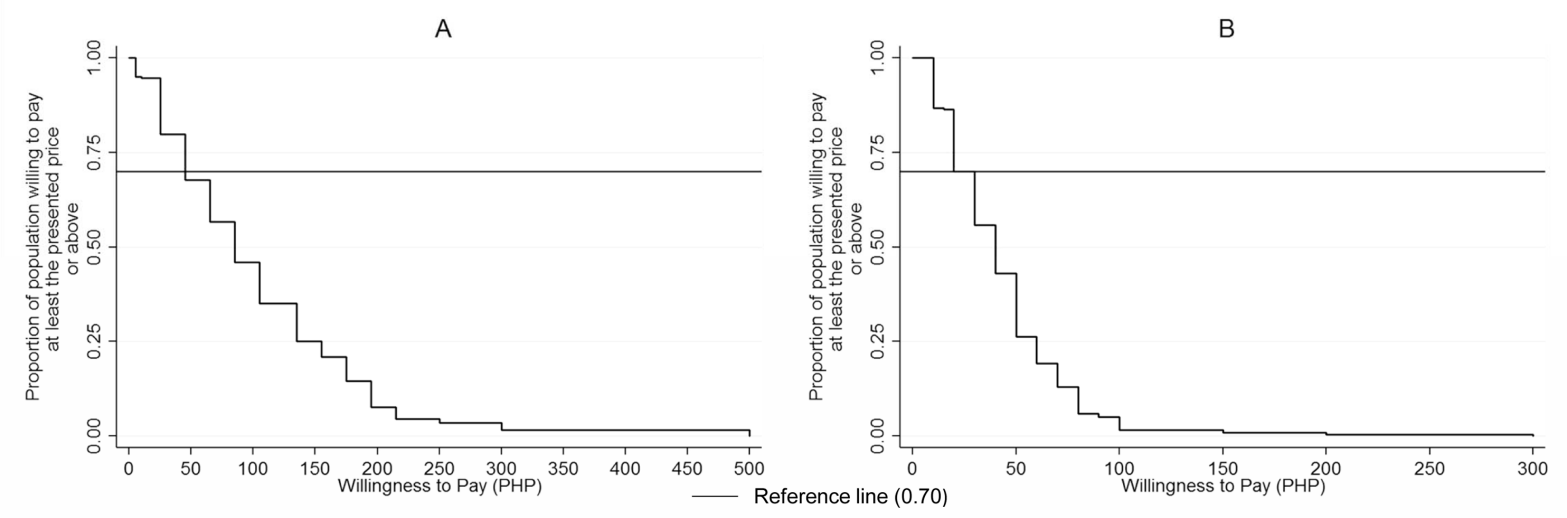
**A. Inverse Demand Curve for WTP for Vaccination.** Relationship between WTP for vaccination price and the proportion of survey population willing to pay at least the presented price in PHP. B. Inverse Demand Curve for WTP for Registration. Relationship between WTP for registration price and the proportion of survey population willing to pay at least the presented price in PHP

Fig 4A and 4B displays the proportion of the population willing to pay a given price or more for dog vaccination and registration. The hypothetical demand for dog vaccination falls gradually as price increases while it falls rapidly for dog registration.

In the univariate analysis, WTP for vaccination was negatively associated with age and positively associated with income. Willingness to pay decreased as age increased and people in the relatively higher income group (above 120,001 PHP and above) were, on average, willing to pay significantly more than people of the relatively lower income group categories (120,000 PHP and below) (Table 4). Also, we observed similar variables and direction of association for WTP for registration as we did for vaccination. In addition to age, gender, income, and municipality of residency, dog ownership status and number of household dogs predicted WTP for dog registration (Table 4).

**Table 4:** Univariate analyses of demographic factors associated with willingness to pay for rabies vaccination and registration, Ilocos Norte, Philippines, August 2012.

	Vaccination		Registration	
Independent variables	Coef.^ᶻ^ (95%CI)	*P*-value	Coef. ^ᶻ^ (95%CI)	*P*-value
**Age**				
20 to 39*				
40 to 64	-19.11(-39.42–1.20)	**0.065**	-13.10(-22.38 –-3.81)	**0.006**
≥ 65	-40.31(-70.74 –-9.88)	**0.009**	-18.76(-29.51 –-8.01)	**0.001**
**Gender**				
Male*				
Female	15.52 (-1.69–32.74)	**0.077**	6.97 (0.73–13.22)	**0.028**
**Employment status**				
Unemployed *				
Employed	8.30 (-16.72–33.33)	0.516	4.80 (-3.67–13.27)	0.267
**Dog ownership status**				
Does not own dog(s) *				
Own dog(s)	-5.82 (-29.00–17.35)	0.623	-10.80 (-18.63 –-2.97)	**0.007**
**Total no. of household members**				
1 to 5*				
6 to 10	-6.97 (-33.78–19.85)	0.611	6.94 (-2.50–16.37)	0.150
≥ 11	5.50 (-73.84–84.85)	0.892	-0.44 (-28.27–27.40)	0.976
**Total number of dogs**				
No dog(s)*				
1 to 3	-7.48 (-33.30–18.34)	0.570	-10.84 (-18.46 –-3.21)	**0.005**
4 to 6	6.08 (-25.11–37.27)	0.703	-13.54 (-28.08–1.00)	**0.068**
≥ 7	-0.75 (-50.50–49.00)	0.977	4.64 (-16.35–25.63)	0.665
**Household Income**				
120,000 and below*				
120,001 and above	38.39 (16.37–60.41)	**0.001**	12.03 (1.77–22.30)	**0.022**
**Municipality of residency**				
Laoag City*				
Marcos	15.32 (-44.27–74.90)	0.614	1.15 (-21.31–23.61)	0.920
Pagudpud	-33.00 (-77.17–11.18)	0.143	-7.97 (-27.51–11.57)	0.424
Paoay	-35.81 (-69.77 –-1.85)	**0.039**	-8.05 (-23.09–7.00)	0.295
Pasuquin	-62.23 (-93.60 –- -30.85)	**<0.001**	-8.83 (-28.07–10.41)	0.369
Piddig	12.95 (-45.97–71.87)	0.667	-2.53 (-23.86–18.80)	0.816
Pinili	-8.01 (-60.72–44.70)	0.766	-6.16 (-26.31–13.99)	0.549
San Nicolas	-22.85 (-59.83–14.13)	0.226	-1.28 (-17.77–15.21)	0.879
Sarrat	-30.33 (-75.49–14.84)	0.188	-3.40 (-24.45–17.65)	0.751
Solsona	30.39 (-17.03–77.81)	0.209	6.70 (-11.35–24.74)	0.467
Vintar	-43.21 (-83.38 –-3.04)	**0.035**	-14.47 (-31.62–2.69)	**0.098**
Bacarra	33.78 (-14.22–81.79)	0.168	2.39 (-14.83–19.61)	0.785
Badoc	-16.20 (-54.64–22.24)	0.409	-2.24 (-18.54–14.04)	0.787
Banna	-32.36 (-76.77–12.06)	0.153	-16.99 (-33.12–0.86)	**0.039**
Batac	4.68 (-32.26–41.62)	0.804	-4.07 (-17.95–9.81)	0.565
Burgos	6.89 (-50.31–64.08)	0.814	20.91 (-6.85–48.67)	0.140
Dingras	-7.32 (-47.63–32.98)	0.722	6.19 (-11.75–24.12)	0.499

ᶻ Differences in mean WTP reported in PHP

* Reference group

Some of the knowledge parameters were also found to be independently associated with participants’ WTP in the univariate analysis (Table 5). Adequate recognition of the outcome of rabies in humans was positively associated with participants’ WTP for dog registration and vaccination, while adequate recognition of rabies signs in dogs and in humans were only associated with participants’ WTP for registration.

**Table 5:** Univariate analysis of knowledge factors associated with the willingness to pay for rabies vaccination and registration, Ilocos Norte, Philippines, August 2012.

Knowledge parameters	Vaccination		Registration	
	Coef. (95%CI)	*P*-value	Coef.(95%CI)	*P*-value
***How does a person get rabies?***				
Don’t know*				
Limited recognitionᵓ	52.99 (35.62–70.36)	<**0.001**ᶠ	14.47 (-24.46–53.41)	0.464
Adequate recognitionᵈ	7.63 (-16.16–31.41)	0.529	5.31 (-1.62–12.24)	0.133
***What happens to MOST people who become ill with rabies?***				
Don’t know *				
Limited recognitionᵐ	38.73 (15.08–62.38)	**0.002**	13.53 (-1.87–28.92)	**0.085**
Adequate recognitionᵑ	27.59 (6.16–49.02)	**0.012**	12.92 (4.64–21.20)	**0.002**
***What are the signs of rabies in persons?***				
Don’t know*				
Limited recognitionᵃ	14.52 (-61.03–90.06)	0.707	13.00 (-20.93–46.93)	0.453
Adequate recognitionᵇ	3.63 (-20.16–27.43)	0.765	7.05 (1.44–12.67)	**0.014**
***What are the signs of rabies in dogs?***				
Don’t know*				
Limited recognitionᵃ	22.38 (-25.25–70.00)	0.357	13.20 (-3.83–30.24)	0.129
Adequate recognitionᵇ	16.39 (-2.21–35.00)	**0.084**	13.26 (5.88–20.63)	**0.001**
**Are you willing to pay the stated amount every year?**				
Yes*				
No	-67.82 (-86.11 –-49.53)	**<0.001**	-28.13 (-33.93 –-22.33)	**<0.001**
Not Sure	40.65 (-28.59–109.90)	0.250	7.26 (-12.02–26.53)	0.461

* Reference group

ᶠ *P*-value based on only two observations.

ᵃThis category comprises of individuals who stated symptoms that are not specific to rabies.

ᵇThese category comprises of individuals who stated at least one rabies related symptom.

ᵓThis category comprises of individuals who stated exposures that are not specific to rabies.

ᵈThese category comprises of individuals who stated at least one rabies related exposure.

ᵐThis category comprises of individuals who stated outcomes that are not specific to clinical rabies.

ᵑThese category comprises of individuals who identified any clinical manifestations of rabies or stated “death” as the clinical outcome of rabies in humans.

Participants’ willingness to commit to pay for each of their dogs, annually was also found to be an important determinant of WTP for vaccination and registration in this study. Those who were not willing to commit to pay each year were, on average, willing to pay significantly less for dog vaccination and registration (Table 5). In assessing the characteristics of participants willing to commit to pay each year in this survey, participants aged 20 to 39 years were 3.94 (1.11–13.98) times as likely to be willing to commit to pay each year as those who were over the age of 65 years. Moreover, we also found that people who stated they strongly liked dogs were 4.22 (1.52–11.75) times as likely to be willing to commit to pay as people who strongly disliked dogs. This indicates that participants of younger age group or participants with a more favorable attitude towards dogs may be willing to commit to pay for each of their dogs annually.

Similar direction and magnitude of association seen in the univariate linear regression were also observed in the multivariable analysis. A number of factors (such as age, income, number of dogs, participants willingness to commit to pay for each of their dogs annually, and participants’ knowledge regarding the signs of rabies in dogs) that were significantly associated with WTP in the univariate analysis remained independently associated with the WTP for dog vaccination and/or registration in the multivariable model. Similarly, age was a significant predictor of the amount individuals were willing to pay for vaccination and registration in the multivariable model as it was in the univariate analysis. We observed that even after adjusting for the other significant determinants of WTP for vaccination/registration, the higher the age, the lower the amount individuals were willing to pay (Table 6).

**Table 6:** Multivariable analysis of factors associated with willingness to pay for rabies vaccination and registration, Ilocos Norte, Philippines, August 2012.

Independent variables	Vaccination		Registration	
	Coef.(95%CI)	*P*-value	Coef.(95%CI)	*P*-value
**Age**				
20 to 39*				
40 to 64	-14.72 (-35.60–6.17)	0.167	-13.49 (-23.13 –-3.85)	**0.006**
≥ 65	-33.09 (-61.13 –-5.05)	**0.021**	-16.86 (-30.65 –-3.06)	**0.017**
**Total number of dogs**				
No dog(s)*				
1 to 3			-11.18 (-19.95 –-2.41)	**0.013**
4 to 6			-18.85 (-30.57 –-7.13)	**0.002**
≥ 7			5.22 (-8.82–19.27)	0.466
**What are the signs of rabies in dogs?**				
Don’t know*				
Limited recognitionᵓ			9.72 (-5.84–25.29)	0.221
Adequate recognitionᵇ			11.14 (3.46–18.83)	**0.005**
**Household Income (PHP)**				
120,000 and below*				
120,001 and above	35.64 (10.05–61.26)	**0.006**		
**Are you willing to pay the stated amount for each of your dog, every year?**				
Yes*				
No	-64.35 (-85.32 –-43.38)	**<0.001**	-24.63 (-32.12 –-17.14)	**<0.001**
Not Sure	28.42 (-55.44–112.56)	0.508	4.90 (-13.48–23.28)	0.601
**Municipality of residency**				
Laoag City*				
Marcos	8.57 (-12.38–29.52)	0.423	5.38 (-0.52–11.30)	0.074
Pagudpud	-29.81 (-51.44 –-8.18)	**0.007**	-15.34 (-21.70 –-8.97)	**<0.001**
Paoay	-25.49 (-46.77 –-4.22)	**0.019**	-3.59 (-24.12–16.93)	0.731
Pasuquin	-63.59 (-85.38 –-41.81)	**<0.001**	-11.42 (-18.70 –-4.14)	**0.002**
Piddig	-1.15 (-23.90–21.59)	0.921	-5.72 (-13.72–2.27)	0.162
Pinili	1.94 (-19.76–23.64)	0.861	-6.48 (-12.22 –- -0.77)	**0.026**
San Nicolas	-26.85 (-50.32 –-3.38)	**0.025**	-0.24 (-7.72–7.24)	0.950
Sarrat	-18.77 (-39.74–2.19)	0.079	8.60 (1.59–15.62)	**0.016**
Solsona	28.10 (3.81–52.40)	**0.023**	6.15 (-0.29–12.58)	0.061
Vintar	-32.64 (-54.07 –-11.21)	**0.003**	-6.64 (-15.62–2.34)	0.147
Bacarra	62.66 (12.05–113.27)	**0.015**	1.25 (-21.29–23.78)	0.914
Badoc	-17.77 (-48.06–12.52)	0.250	-4.23 (-10.21–1.74)	0.165
Banna	-12.87 (-35.97–10.23)	0.275	-7.41 (-16.14 –- 1.33)	0.096
Batac	8.79 (-12.10–29.68)	0.410	-2.12 (-8.95–4.72)	0.544
Burgos	-64.81 (-84.100 –-44.62)	**<0.001**	15.88 (5.57–26.19)	**0.002**
Dingras	-5.65 (-46.52–35.23)	0.787	0.36 (-13.95–14.67)	0.961
R^2^		**0.2792**		**0.2745**

No direct relationship was observed between WTP and employment status. However, we found a strong association between age and employment status. Specifically, those who were between the ages of 20–39 years, and 40–64 years were 11.20 (CI, 4.74 to 26.47) and 11.37 (CI, 5.25 to 24.61) times more likely to be employed, respectively, as those who were over the age of 65. Further stratification of data by employment status revealed that there was no statistically significant relationship between age and WTP for vaccination in the employed group while in the unemployed group, the relationship between age and WTP for vaccination was still significant. In the case of dog registration, however, the association between age and WTP still persisted regardless of employment status. Therefore employment status may have modified the relationship between age and individuals’ WTP for vaccination, but may have had no effect on individuals’ WTP for registration.

## Discussion

In the present study, we elucidate the maximum amount residents of Ilocos Norte, Philippines were willing to pay for dog vaccination and registration. On average, Ilocos Norte residents were willing to pay 69.65 PHP (approximately 1.67 USD) for dog vaccination and 29.13PHP (0.70 USD) for dog registration. Eighty-six per cent of respondents were willing to pay the stated amount to vaccinate each of their dogs, annually. The study findings give policy makers some indication of how much residents may be willing to contribute financially towards dog vaccination and registration in this community.

In recent years, actual registration and vaccination fees have successfully been introduced in other parts of the country as well as in another rabies endemic countries [10, 28]. In Bohol, for instance, dog vaccination and registration fees charged to dog owners were, on average, 75.49 PHP (approximately 1.74 USD) and 50 PHP (approximately 1.11 USD in 2009), respectively [9, 10]. These charged fees were slightly higher than what was found in the present study which implies that Bohol residents may attach higher value towards dog vaccination and registration compared to Ilocos Norte residents. This may have resulted from the implementation of the Bohol Rabies Prevention and Elimination Program which may have increased rabies awareness as well as promoted community level participation, hence enhanced commitment [10]. The observed difference may also have resulted from the hypothetical nature of this survey, which uses individuals’ stated preference in Ilocos Norte as opposed to practical experience in Bohol.

The average stated maximum WTP for vaccination, in general, was found to be higher than the overall estimated per dog vaccination cost for most Asian, African, and Latin American countries (1.55 USD) [3]. In comparison to specific rabies endemic countries, the WTP value was found to exceed the estimated per dog vaccination cost in Thailand (0.52 USD) while it was lower than the cost found in Tanzania (1.73 USD & 5.55 USD per dog vaccinated in two different settings using two different vaccination strategies) and N’Djamena, Chad (found through both owner-charged (19.40 USD) and free vaccination campaigns (≈2.90–3.80 USD)) [3, 29–32].

The result of our study, however, suggested that the stated maximum WTP for dog vaccination in this population may not pay for the entire cost of a vaccination program if 70% vaccination coverage of the estimated dog population is to be attained. The estimated program cost per vaccinated dog in the Philippines varies from one locality to another. For example, the estimated cost per dog vaccinated in Muntinlupa City during 1991 was 0.78 USD (≈1.11 USD in 2012) while in Bohol Island it was estimated at 1.62 USD, excluding the manpower cost [10, 33]. These costs were estimated for vaccination coverage greater than 70% attained in these locations. The cost estimated in Bohol, which geographically is more comparable to Ilocos Norte than Muntinlupa City, is higher than the stated maximum WTP for vaccination found among most of the participants in our study. Only 46% of our survey participants had a stated willingness to pay for dog vaccination of 1.55 USD or higher. Theoretically, if the stated WTP holds true, the price of dog vaccination should be set between 25–45 PHP (0.59–1.08 USD) to ensure that 68%-79% of our study participants would be willing to pay for dog vaccination. Practically, however, stated WTP and observed pay for dog vaccination has been found to vary considerably across price of vaccination charged to owners [28]. Therefore, we may expect some degree of variation between stated and actual pay.

Participants who were willing to pay for dog vaccination were significantly more likely to be willing to pay for dog registration. This may be because dog registration fees are mandatory by law in the Philippines which may have influenced their decision to accept these fees as obligatory fees. Another explanation for their willingness to pay for registration might be the fact that registration fees act as insurance to receive subsidized post-exposure treatments from the government if dog owners are bitten by a dog [34].

In this study, we found that dog owners were willing to pay significantly less for both registration and vaccination services than those who did not own dogs (Table 4). This discrepancy in WTP may have occurred simply because people who did not own dog/s may not have considered the practical economic implications/consequence of paying compared to those who actually utilize these services, resulting in a significant variation in WTP between these groups.

The present study also found that WTP for dog vaccination and registration was influenced by some of the demographic factors and pre-existing knowledge. Among these factors, we found that age, income, and participants’ willingness to commit to pay each year, and municipality of residency were significantly associated with WTP.

Age was an important determinant of WTP and was strongly associated with both WTP for dog vaccination and registration even after controlling for participants’ income (in the case of WTP for dog vaccination), municipality of residency, willingness to pay for each of their dogs annually, and number of dogs owned (in the case of WTP for dog registration). This, in part, may be explained by the relationship that was observed between age and employment status in this study. Although there was no statistically significant relationship between employment status and WTP in this study, the strong relationship observed between WTP and age, and the great dependency of employment status on age of individuals may have implications not on their WTP, but rather on their ability to pay. This, in turn, may give cause for concern about relying on a single strategy of collecting dog rabies vaccination fees and could be used to argue that strategies to shield the elderly (who may be unemployed) from user fees related to dog vaccination need to be considered in the implementation of such programs.

Another explanation for the strong association between WTP and age in this survey may be because of the relationship observed between age and rabies awareness. Specifically, compared to those younger than 65 years old, participants 65 years old and over were less likely to have heard about rabies, and less likely to have correctly identified the outcome of rabies in humans. This gap in rabies awareness that exist among the elderly may also have had an indirect effect on their WTP. This may be addressed by implementing educational campaigns that target individuals in this age category. Raising rabies awareness among the younger generation may also act as a long term solution.

The positive association between income level and the amount individuals were willing to pay for dog vaccination and registration was consistent with the theoretical construct of positive income elasticity which states that higher income should be associated with higher WTP. However, this again may imply that people of the lower income category may be less able to pay than people of high income groups. Therefore there may be a need for adjusting premiums for those within the low income category through subsidization in order to balance participation.

This study also found that WTP was significantly influenced by individuals’ willingness to commit to pay for dog vaccination and registration. Ages 20–39 years and/or having favorable attitude towards dogs partially defined the characteristics of those who were willing to commit to pay. This may suggest that programs that enhance favorable attitude towards dogs, targeting particularly those of the younger age groups (20 to 39 years) may be a good strategy to attain a continued financial commitment that is required to sustain canine rabies elimination programs such as mass dog vaccination and registration campaigns.

Participants with higher knowledge of the outcome of rabies in humans were willing to pay a significantly higher amount than those who had no knowledge. This positive association may imply that more individuals’ awareness of rabies outcome in humans may mean more value towards dog vaccination and registration. However, as rabies cases fall, awareness is likely to fall and in turn may affect WTP.

There are some limitations of the present study that need to be considered. In the use of contingent valuation technique, possible biases which may distort actual from hypothetical WTP may arise [17]. Reliability upon this technique highly depends on the information respondents possess of the service being valued. Given that the service being valued in this study is dog rabies vaccination and registration, and that participants have demonstrated some degree of awareness about them, the WTP value is likely to be valid and reliable. Second, starting point bias may influence the validity of stated WTP estimates. However, strategies were developed to minimize this bias by increasing the number of bidding levels in addition to randomly assigning the bids to participants. Third, this survey utilized a convenient sampling strategy to determine participating households and participants. This factor may limit the external validity/ generalizability of the survey. In the effort to maximize generalizability, the survey attempted to improve representativeness through maximizing the number of cluster within each municipality as well as increasing the number of household selected within each cluster. Fourth and last, the utilization of paper-based questionnaires may by itself carry the risk of introducing bias to the study. To prevent biases related to questionnaire studies from being introduced, this study used in-person interview strategy which provided an opportunity for participants to inquire and request for more clarification if uncertain about specific questions. In addition, enumerators were also pre-trained in the survey methods and questionnaires were translated into the local language in order to maintain consistency and improve comprehension. As a result of this effort, no strategically introduced bias was observed and missing values were seen to be randomly distributed across respondents and enumerators. The analysis has also accounted for these missing values to eliminate errors that could result from miscalculation.

### Conclusion

This study provided evidence on the perceived monetary value of dog vaccination and registration in Ilocos Norte, Philippines by assessing the maximum amount of money individuals are willing to pay. It found that the majority of Ilocos Norte residents stated they were willing to pay an average of 1.67 USD for dog vaccination and 0.70 USD for dog registration. Socio-economic and demographic factors such as age, income, and number of dogs owned, municipality of residency, and participants willingness to pay for each of their dogs annually were found to influenced stated WTP. This factors, therefore, may need to be considered prior to the introduction of such fees to the public. Creating rabies awareness and promoting favorable attitude towards dogs may also aid in the effective delivery of such programs.

## Supporting Information

S1 File Survey questionnaire. (DOCX)

S2 File Survey data. (XLSX)
