# Supplementary material for: Willingness to Pay for Dog Rabies Vaccine and Registration in Ilocos Norte, Philippines (2012)
Source: PLoS Negl Trop Dis. 2016 Mar 21;10(3):e0004486. doi: 10.1371/journal.pntd.0004486 (PMC4801174; doi:10.1371/journal.pntd.0004486)
Supplement: S1 File — (DOCX) [file pntd.0004486.s001.docx]

**Form 1: HOUSEHOLD QUESTIONNAIRE**

*(Complete one form for each household. Fill the blanks or mark the answers.)*

**SECTION 1. Site Information**

**Cluster no.: ______ House ID no.: _____ Unique Study ID: _____-_____**

Street/Purok: ___________________ Barangay: __________________ Municipality: __________________

Interview Date: _______/_______/_________ Interviewer Initials: __________ Translator Initials: ________

Day Month Year Time of Interview: _____________ to ______________

**SECTION 2. Household Dog Information**

1. Does the household currently own dogs? Adda kadi aso a taraken ti pamilya iti agdama? (***If “No” Skip to question 1.9***) (No AWAN, mapan iti Saludsod No. 1.9)

❒ NO AWAN ❒ YES ADDA ❒ DON’T KNOW DIAK AMMO

If **YES**, No **ADDA**,

- 1. How many dogs does the household currently own? Mano ti tarakenyo nga aso ita?_______

Total number of male dogs Mano ti lalaki nga aso _____________

Total number of female dogs Mano ti aso a babai ____________

- 1. How many dogs were newly obtained in the past 12 months.? Mano ti asoyo a nainayon iti uneg ti 12 a bulan?____
  2. How old is/are the dog(s)? Mano ti tawen ti/dagiti asoyo?

1. ____
2. ____
3. ____
4. ____
5. ____
6. ____
   1. Has your dog been vaccinated for rabies in the past 2 years? Nabakunaan kadi ti asoyo iti naglabas a 2 a tawen? (if answer is “No” or “Don’t know”, skip to question #1.8) (No ti sungbat ket **SAAN** wenno **DIAK AMMO**, mapan iti Saludsod 1.8)

❒ NO SAAN ❒ YES WEN ❒ DON’T KNOW DIAK AMMO

*If* ***YES No WEN***

- 1. If your dog was vaccinated for rabies in the past 2 years, how many times have your dog(s) been vaccinated? No nabakunaan ti asoyo iti naglabas a 2 a tawen, mano daras a nabakunaan?

1. One time Naminsan b. Two times Namindua
2. More than two times Ad-adua ngem namindua d. Don’t know Diak ammo
   1. If your dog was vaccinated for rabies in the past 2 years, how old was your dog when it received the vaccination for the first time? No nabakunaan ti asoyo iti naglabas a 2 tawen, ana edad na idi damo a mabakunaan? _____________________
   2. If your dog was vaccinated in the past 2 years, have you ever paid for having your dog(s) vaccinated? No ti asoyo ket nabakunaan iti naglabas a 2 tawen, nagbayad kayo kadi iti pannakabakunada? (If “No” or “Don’t know” skip to question #1.8) (No SAAN wenno DIAK AMMO, mapan iti Saludsod 1.8)

❒ NO SAAN ❒ YES WEN ❒ DON’T KNOW DIAK AMMO

- - 1. If you have paid for having your dog(s) vaccinated before, for which service were you charged for? No nagbayad kayo iti pannakabakuna ti asoyo, para ana ti inbayadyo? (circle all that apply) (bilogen ti umno a sungbat)
       - 1. Vaccine fee Para bakuna
         2. Vaccine supply fee (needles, alcohol, swab, etc) Para gamit panagbakuna (iringgilya, alcohol, kapas, ken dadduma pay)
         3. Dog registration fee Para rehistro ti aso
         4. Shot administration fee (overhead) Para iti serbisyo
         5. Other Dadduma pay
         6. Don’t know Diak ammo

1.7.1.1 If the service you were charged for is “other”, please specify the type of service you were charged for? No ti bayad ket DADDUMA PAY, pakilanad no ti para ana ti binayadanyo.

- - 1. _____________________________________
    2. If you have paid for having your dog(s) vaccinated before, please specify the approximate amount of money you were charged per dog in *pesos* (PHP)? No nagbayad kayo iti pannakabakuna ti asoyo, pakilanad no mano pisos ti binayadanyo ti kada aso?
       - 1. ___________________
  1. Do you plan to have your dog(s) vaccinated in the future? Adda planoyo nga agpabakuna kadagiti asoyo iti masakbayan?

❒ NO AWAN ❒ YES ADDA ❒ DON’T KNOW DIAK AMMO

(***Interviewer: Ask permission to see the dog/s to assess the dog condition. Use form 2***) (Para iti agsalsaludsod, dawaten ti permiso a makita dagiti aso tapno makita ti kondisyonda. Usaren ti *Form*2)

If **NO** No **AWAN**

- 1. If the household does NOT currently own dogs*,* has anyone in the household owned a dog in the past two years? No ti pamilya ket AWAN asoda iti agdama, adda kadin nakapadas ti nagtaraken iti aso iti uneg ti naglabas a 2 tawen?

❒ NO AWAN ❒ YES ADDA ❒ DON’T KNOW DIAK AMMO

- 1. *If no,* has anyone in the household *ever* owned a dog? No AWAN, adda kadin miyembro ti pamilya a nagtarakenen iti aso?

❒ NO AWAN ❒ YES ADDA ❒ DON’T KNOW DIAK AMMO

- 1. Does anyone in the household plan to own a dog in the near future? Adda kadi miyembro ti pamilya nga kayatna agtaraken iti aso iti masakbayan?

❒ NO AWAN ❒ YES ADDA ❒ DON’T KNOW DIAK AMMO

1. Are there unowned dogs in your neighbourhood? Adda kadi aso nga awan makintaraken iti kaarrubayo?

❒ NO AWAN ❒ YES ADDA ❒ DON’T KNOW DIAK AMMO

- 1. If **YES** (Give average numbers) No **ADDA** (Pattapattaen no mano)

a. How many are always in the community? Mano ti adda a kanayon iti komunidad? ____

b. How many are unidentified dogs? Mano ti saanyo ammo no asino akin-aso?____

1. Do you care for any other dog than your own in the community? Tamtamingenyo kadi dagiti aso iti komunidad?

❒ NO SAAN ❒ YES WEN ❒ DON’T KNOW DIAK AMMO

- 1. If **YES***,* how many other dogs do you care for? No **WEN**, mano ti saanyo nga aso ti tartaraknenyo?____________
  2. What level of care do you provide for the community dog(s)? Check all that apply. Ana ti it-itedyo a panangtaraken kadagiti aso ti komunidad?

1. None Awan
2. Food Makan
3. Water Danum
4. Shelter Paglinungan
5. Veterinary Care (*for this option, the interviewer should ask whether if the individual has ever taken a community dog to a veterinarian*)

Serbisyo ti beterinaryo (no daytoy ti sungbat, ti parasaludsod ket damagenna no napadasanen ti pagsalsaludsodan ti nangipan iti aso ti komunidad iti beterinaryo)

1. Other Sabali pay
2. Declined to answer Agtukiad a sumungbat
   - 1. If the level of care you provide for the community dog(s) is *“Other*”, please specify the level of care No ti panangtaripato a mait-ited kadagiti aso ti komunidad ket SABALI PAY, pakilanad no ana a panangtaripato.
3. *_______________________________________*

**SECTION 3: Information on Willingness to Pay for Dog Vaccination and Registration**

**Introductory statement**

*(To be read to the interviewee prior to presenting the biding game)*

As you might be aware, dog vaccination and registration have been made available to this community in the past to prevent the spread of a dangerous disease that can be transmitted from animals to humans. To continue this effort, it may be necessary to introduce dog registration and vaccination fees to the community in the near future. Accordingly, in the next section, we will be asking a series of questions that will enable us to understand how much you are able and willing to pay for dog registration and vaccination. Please answer the question as sincerely as possible because the provision of the service will highly depend on the true amount you are able and willing to pay for these services.

**Directions for Interviewer**:

*The next two subsections (3.1 and 3.2) will assess individual’s Willingness to Pay (WTP) for dog vaccination and registration fee. This will be presented as a* ***bidding game.*** *To assess WTP for vaccination fee****,*** *the participants should first be offered an initial hypothetical maximum price for dog rabies vaccine of 105 PHP (approximately $2.50) (question 4). If the respondent accepts or answers “Yes” to this initial price, he/she should be directed to question 5 and should continuously be offered higher and higher price step by step until the respondent rejects or says no. Alternatively, if the respondent rejects (“No”) the initial price (question 4), then the respondent should be directed to question 6 and the prices should repeatedly be decreased step by step until the respondent accepts a given price or reaches zero. The same methodology will be used to assess Willingness to Pay for dog registration fee (starting from Question 8 to 11).*

**SUB-SECTION 3.1 : WTP for dog vaccination**

1. In the future, would you continue paying or be willing and able to pay *105 PHP* **for vaccination**? Iti masakbayan, kayatyo kadi a maituloy wenno umanamongkayo ken kabaelanyo ti agbayad iti 105PhP para bakuna?

**a. Yes** (If “Yes” please continue to **question 5**) **WEN** (No WEN, mapan iti Saludsod 5)

**b. No** (If “No” please continue to **question 6**) **SAAN** (No SAAN, mapan iti Saludsod 6)

*(If* ***YES****) (No* ***WEN****)*

1. Would you be willing and able to pay 135 PHP? Umanamong kayo kadi nga agbayad iti 135 PhP?
2. Yes Wen b. No Saan
   - *(If answered “Yes” to the above) would you be willing and able to pay 155 PHP? (No ti sungbat ket WEN iti ngato) umanamong ken kabaelanyo kadi nga agbayad iti 155 PhP?*
     - - 1. Yes Wen b. No Saan
   - *(If answered “Yes” to the above) would you be willing and able to pay 175 PHP? (No ti sungbat ket WEN iti ngato) umanamong ken kabaelanyo kadi nga agbayad iti 175 PhP?*
     - - 1. Yes Wen b. No Saan
   - *(If answered “Yes” to the above) would you be willing and able to pay 195 PHP? (No ti sungbat ket WEN iti ngato) umanamong ken kabaelanyo kadi nga agbayad iti 175 PhP?*
     - - 1. Yes Wen b. No Saan
   - *(If answered “Yes” to the above) would you be willing and able to pay 215 PHP? (No ti sungbat ket WEN iti ngato) umanamong ken kabaelanyo kadi nga agbayad iti 215 PhP?*
     - - 1. Yes Wen b. No Saan
   1. If you are able and willing to pay more than 215PhP, please specify the amount you are willing to pay. No kabaelan ken umanamong kayo nga agbayad iti nangatngato ngem 215PhP, pakilanad no mano ti kayatyo a bayadan.
3. __________________________
   1. Are you willing to pay this fee for each of your dogs every year? Umanamongkayo met lang nga agbayad iti daytoy a kantidad iti kada asoyo iti tinawen?

❒ NO SAAN ❒ YES WEN ❒ DON’T KNOW DIAK AMMO

*(If* ***NO****) (no* ***SAAN****)*

1. Would you be willing and able to pay 85 PHP? Umanamong ken kabaelanyo kadi nga agbayad iti 85 PhP?
   - 1. Yes Wen b. No Saan
   - *(If answered “No” to the above)* would you be willing and able to pay 65 PHP? *(No ti sungbat ket SAAN iti ngato) umanamong ken kabaelanyo kadi nga agbayad iti 65 PhP?*
     1. Yes Wen b. No Saan
   - *(If answered” No” to the above) would you be willing and able to pay 45 PHP? (No ti sungbat ket SAAN iti ngato) umanamong ken kabaelanyo kadi nga agbayad iti 45 PhP?*
     1. Yes Wen b. No Saan
   - *(If answered” No” to the above) would you be willing and able to pay 25 PHP? (No ti sungbat ket SAAN iti ngato) umanamong ken kabaelanyo kadi nga agbayad iti 25 PhP?*
     1. Yes Wen b. No Saan
   - *(If answered” No” to the above) would you be willing and able to pay 5 PHP? (No ti sungbat ket SAAN iti ngato) umanamong ken kabaelanyo kadi nga agbayad iti 5 PhP?*
     1. Yes Wen b. No Saan
   - *(If answered” No” to the above) are you willing and able to pay $0.00 PHP? (No ti sungbat ket SAAN iti ngato) umanamong ken kabaelanyo kadi nga agbayad iti $0.00 PhP?*
     1. Yes Wen b. No Saan
2. Are you willing to pay this fee for each of your dogs every year? Umanamongkayo kadi nga agbayad iti kada asoyo iti kada tawen?

❒ NO SAAN ❒ YES WEN ❒ DON’T KNOW DIAK AMMO

**SECTION 3.2: WTP for dog registration**

1. In the future, would you continue paying or be willing and able to pay *40 PHP* for dog registration? Iti masakbayan, siaannugot kayo kadi wenno umanamong ken kabaelanyo ti agbayad iti 40PhP para iti pannakarehistro dagiti asoyo?

**a. Yes** (If “Yes” please continue to **question 9**) **Wen** (No WEN, mapan iti Saludsod 9)

**b. No** (If “No” please continue to **question 10**) **Saan** (No SAAN, mapan iti Saludsod 10)

*(If* ***YES****) (No* ***WEN****)*

1. Would you be willing and able to pay 50 PHP? Umanamong kayo kadi nga agbayad iti 50 PhP?
   - 1. Yes Wen b. No Saan
   - *(If answered “Yes” to the above) would you be willing and able to pay 60 PHP? (No ti sungbat ket WEN iti ngato) umanamong ken kabaelanyo kadi nga agbayad iti 60 PhP?*
     1. Yes Wen b. No Saan
   - *(If answered “Yes” to the above) would you be willing and able to pay 70 PHP? (No ti sungbat ket WEN iti ngato) umanamong ken kabaelanyo kadi nga agbayad iti 70 PhP?*
     1. Yes Wen b. No Saan
   - *(If answered “Yes” to the above) would you be willing and able to pay 80 PHP? (No ti sungbat ket WEN iti ngato) umanamong kayo kadi nga agbayad iti 80 PhP?*
     1. Yes Wen b. No Saan
   1. *If you are able and willing to pay more than 80PHP, please specify the amount you are willing to pay.* No kabaelan ken umanamong kayo nga agbayad iti nangatngato ngem 80PhP, pakilanad no mano ti kayatyo a bayadan.
      - - 1. *__________________________*

*(If* ***NO****) (no* ***SAAN****)*

1. Would you be willing and able to pay 30 PHP? ? Umanamong ken kabaelanyo kadi nga agbayad iti 30 PhP?
   - 1. Yes Wen b. No Saan
   - *(If answered “No” to the above)* would you be willing and able to pay 20 PHP? *(No ti sungbat ket SAAN iti ngato) umanamong ken kabaelanyo kadi nga agbayad iti 20 PhP?*
     1. Yes Wen b. No Saan
   - *(If answered” No” to the above) would you be willing and able to pay 10 PHP? (No ti sungbat ket SAAN iti ngato) umanamong ken kabaelanyo kadi nga agbayad iti 10 PhP?*
     1. Yes Wen b. No Saan
   - *(If answered” No” to the above) would you be willing and able to pay 0 PHP? (No ti sungbat ket SAAN iti ngato) umanamong ken kabaelanyo kadi nga agbayad iti 0 PhP?*
     1. Yes Wen b. No Saan

**SECTION 5. Household Information**

1. Interviewee Sex: ❒ Male Lalaki ❒ Female Babai
2. Interviewee Age: Tawen/Edad ______
3. Interviewee Religion: Relihiyon ______
4. Interviewee’s relationship to the Head of Household? Kapin-ano ti mangidaulo ti pamilya?
   - Self (Head of Household) Isu mismo
   - Spouse Asawa
   - Child Anak
   - Other Relative Sabali a kabagyan

❒ Other Non-relative Sabali a saan a kabagyan

- 1. *If Other,* what is your relationship to Head of Household? *No SABALI, kapin-ano ti mangidaulo ti pamilya?* ________________

1. Is the Head of Household employed? Mangmangged ti mangidaulo ti pamilya?

❒ NO SAAN ❒ YES WEN ❒ DON’T KNOW DIAK AMMO

- 1. *If* ***YES****,* what occupation? *No WEN, ana ti panggedanna?*_______________________________

1. What is the total number of household members at this time: Mano kayo a miyembro ti pamilya iti agdama? ________
   1. How many of them are zero to 5 yrs old? Mano kanyayo ti agtawen 0 inggana 5?_______
   2. How many of them are 6 to 10 yrs old? Manokanyayo ti agtawen 6 inggana 10?_______
   3. How many of them are 11 to 15 yrs old? Manokanyayo ti agtawen 11 inggana 15?_______
   4. How many of them are 16 to 20 yrs old? Mano kanyayoti agtawen 16 inggana 20?_______
   5. How many of them are 21 to 25 yrs old? Mano kanyayo ti agtawen 21 inggana 25?_______
   6. How many of them are 26 to 30 yrs old? Mano kanyayo ti agtawen 26 inggana 30?_______
   7. How many of them are 31 to 35 yrs old? Mano kanyayo ti agtawen 31 inggana 35?_______
   8. How many of them are 36 to 40 yrs old? Mano kanyayo ti agtawen 36 inggana 40?_______
   9. How many of them are 41 to 45 yrs old? Mano kanyayo ti agtawen 41 inggana 45?_______
   10. How many of them are 46 to 50 yrs old? Mano kanyayo ti agtawen 46 inggana 50?_______
   11. How many of them are 51 to 60 yrs old? Mano kanyayo ti agtawen 51 inggana 60?_______
   12. How many of them are 61 to 65 yrs old? Mano kanyayo ti agtawen 61 inggana 65?_______
   13. How many of them are 66 and over? Mano kanyayo ti agtawen 66 agpangato?_______
2. How many are: Mano ti:

Primary school graduate/s? Nagturpos iti elementarya?_____

Primary school undergraduate/s? Saan nakaturpos iti elementarya _____

Secondary school graduate/s? Nagturpos iti sekundarya? _____

Secondary school undergraduate/s? Saan nakaturpos iti sekundarya? _____

College graduate/s? Nagturpos iti kolehiyo _____

College undergraduate? Saan nakaturpos iti kolehiyo? _____

Postgraduate? Nagturpos iti masteral/doctorate? _____

Don’t Know Diak ammo _____

1. How much is the household’s annual income? Mano ti sapul ti pamilya iti uneg makatawen?
   - PhP 120,000 and below
   - Php 120, 001 – 180,000 ❒ PhP 300,001 – 360,000
   - PhP 180,001 – 240,000 ❒ PhP 360,001 – 420,000
   - PhP 240,001 – 300,000 ❒ PhP 420,001 and above

**SECTION 7. Rabies Knowledge & Attitudes Information**

1. Have you ever heard of a disease called rabies? Nangnanggegyo kadin ti rabis?

❒ NO SAAN ❒ YES WEN ❒ DON’T KNOW DIAK AMMO

***(If NO, skip to Question 33)(No SAAN, mapan iti Saludsod 33)***

- 1. If **YES**, from which medium did you get your information on rabies? No **WEN,** kasanoyo a naamuan?
- TV TV ❒ Barangay Assembly Asemblya ti barangay
- Radio Radyo ❒ Poster / Leaflet Poster wenno leaflet
- Newspaper Dyaryo ❒ Others, pls. specify: Sabali pay, pakilanad_______________

1. What are the signs of rabies in a dog? (*list all)* *(selection of common responses was added after pre-test)* Ania dagiti senyales ti rabis iti aso? *(ilista amin)*

❒Fever ❒ Apprehensive watchful look

❒ Afraid of water ❒ Unprovoked aggressiveness

❒ Restlessness ❒ Loss appetite

❒ Aimless running ❒ Depression

❒ Drooling of saliva ❒ Paralysis (Hindleg/Jaw/Tongue)

❒ Quarrelling with other dogs ❒ Others, specify: _____________

1. If you had a dog with any of these signs, what would you do? No adda asoyo nga agpakpakita iti aniaman kadagitoy a senyales, ania ti aramidenyo?

❒ Consult a private vet Agpakonsulta iti pribado a beterinaryo

❒ Consult a government vet or technician Agpakonsulta iti beterinaryo ti gobyerno/teknisyan ti gobyerno

❒ Consult a barangay official Agpakonsulta iti opisyal ti barangay

❒ Don’t know Diak ammo

❒ Nothing Awan

❒ Others, pls. specify: Sabali pay. Pakilanad._____________________________________

1. Do you know how a person gets rabies? Ammoyo kadi no kasano a maaddaan rabis iti tao?

❒ NO SAAN ❒ YES WEN ❒ NOT SURE SAAN SIGURADO

- 1. *If* **YES***,* how does a person get rabies? *No* ***WEN***, kasano a maaddaan iti rabis ti tao?

1. What are the signs of rabies in a person? *(list all)*  *(selection of common responses was added after pre-test)* Ania dagiti senyales ti rabis iti tao? *(ilista amin)*

❒ Afraid of water ❒ Salivation

❒ Afraid of air ❒ Wild/Crazy

❒ Afraid of light ❒ Infection of the wound bite

❒ Apprehensive watchful look (Glaring eyes) ❒ Depressed

❒ Fever ❒ Trembling

❒ Others, specify:_______________ Sabali pay, ilanad:_______________

1. Have you ever known anyone who had rabies? Adda kadi am-ammoyo a na-addaan rabis?

❒ NO SAAN ❒ YES WEN ❒ DON’T KNOW DIAK AMMO

1. What happens to **MOST** people who become ill with rabies? (please make **ONE** answer) Ana ti masansan a mapasamak iti KAADUAN a tao nga agsakit iti rabis? (paki-tsek ti MAYSA lang a sungbat)

❒ Death Matay

❒ No hospital stay, but need a doctor visit Saan a maiospital ngem kasapulan ti pannakabisita ti doktor

❒ Recovery after a hospital stay Agimbag kalpasan maiospital

❒ Resolve without a doctor visit Agimbag a saan a kasapulan ti doktor

❒ Other_______________ Sabali pay

❒ Don’t know Saan nga ammo

- 1. *If Other,* please specify what happens to **MOST** people who become ill with rabies *No SABALI PAY, pakilanad no ana ti mapasamak iti KAADUAN a tao nga agsakit ti rabis)*

*­­­­­­­­­­­­­­­­­* ­­­­­­­­­­a. _________________________________________

**SECTION 8. Responsible Pet Ownership Information**

1. Kindly rate your agreement on the following statements provided below.

**I like dogs very much**

❒Strongly Disagree (Saanak nga umanamong)

❒Slightly Disagree (Saanak unay umanamong)

❒Unsure (Saanak a sigurado)

❒Slightly Agree (Umana-mongak bassit)

❒Strongly Agree (Umana-mongak unay)

**Appendix 2 : Adult Consent Form (Age 21 or over)**

**ADULT (21 YRS AND OVER) CONSENT FORM (ENGLISH):**

**NATAENGAN (EDAD 21 AGPANGATO) PORMA TI PANANGAKLON (ILOCANO):**

**Flesch-Kincaid reading level: 6.6**

**Flesch-Kincaid antas ti panagbasa: 6.6**

**Willingness to Pay for Canine Rabies Vaccination, Philippines**

**Iyaanamong ti Panagbayad Para Bakuna ti Aso Kontra Rabis, Pilipinas**

**WHY IS THIS STUDY BEING DONE?**

**APAY MAARAMID DAYTOY A PANAGSALIKSIK?**

The Centers for Disease Control and Prevention, Atlanta, USA (CDC) and the Ministry of Health of the Philippines is researching diseases that are transmitted by animals to people. We are asking you to be in a research survey.

Dagiti Centers for Disease Control and Prevention, Atlanta, USA (CDC) ken ti Departamento ti Salun-at ti Pilipinas ket agsalsaliksik kadagiti saksakit a mabalin a maiyakar manipud animales iti tao.

**WHAT WILL HAPPEN IN THIS STUDY?**

**ANA TI MAPASAMAK ITI DAYTOY A PANAGSALIKSIK?**

- We will interview you about your knowledge, attitude and practice towards rabies and your valuation of acceptable cost for canine rabies vaccines and dog registration fees.
- Agsaludsod kami kadakayo maipanggep iti ammo, kaugalian, ken aramid maipanggep iti rabis ken ti mabalin ken kaya a kantidad ti bakuna kontra rabis ken pannakarehistro dagiti aso.

If you choose to be in this study:

No kayatyo ti mairaman iti daytoy a panagsaliksik:

- we will ask you questions for about 40 minutes.
- Agsaludsod kami kadakayo iti uneg ti 40 minutos.

**WHAT ARE THE RISKS?**

**ANA DAGITI RISGO?**

There are minimal risks for being in the study. We are only asking for information about your views, and knowledge. Your participation is completely voluntary.

Adda bassit a risgo iti pannakiramanyo iti daytoy a panagsaliksik. Agsaludsod kami iti impormasyon maipanggep iti kapanunotanyo, ken ammoyo. Ti pannakipartisiparyo ket pulos a bulontaryo.

**WILL ANYTHING GOOD HAPPEN TO YOU?**

**ADDA KADI NAPINTAS A MAPASAMAK KANYAYO?**

You may not get any direct benefit from being in this study, but you will help us know more about the valuation of dog vaccine in your community. Information obtained from this study may help the Ministry of Health prevent and treat illnesses caused by animals, particularly in your community.

Mabalin nga awan ti direkta a masagrapyo manipud iti daytoy a panagsaliksik, ngem matulongan dakami no ana ti kabaelanyo para iti bakuna ti aso ditoy lugaryo. Dagiti impormasyon a maadaw iti daytoy a panagsaliksik ket makatulong iti Departamento ti Salun-at para iti pannakaprebentar ken panagagas iti saksakit a gapuanan dagiti animales, aglalo ditoy lugaryo.

**WHAT ABOUT PRIVACY?**

**KASANO MET ITI KINAPRIBADO?**

The information we collect in this study is confidential. To protect your privacy, we will not ask for your name. All the information collected in this project will be kept in locked computer files. Only the CDC persons involved in the survey can view your responses.

Dagiti impormasyon a maadawmi manipud iti daytoy a panagsaliksik ket konpidensyal. Tapno maproteheran ti kinapribadoyo, saanmi a saludsoden ti naganyo. Amin nga impormasyon a maadaw iti daytoy a proyekto ket maidulin iti natulbekan a dokumento iti kompyuter. Dagiti laeng representante ti CDC a kadua iti daytoy a panagsalsaludsod ti makakita dagiti sungbatyo.

**PAYMENT FOR YOUR TIME AND EFFORT**

**BAYAD PARA ITI ORAS KEN BANNOGYO**

You will not receive any payment for being in this study.

Awan ti maawatyo kas bayad iti daytoy a panagsaliksik.

**IF YOU HAVE QUESTIONS**

**NO ADDA MAN SALUDSODYO**

Please call Dr. Loida Valenzuela (+639189337255) from the Provincial Veterinary Office-Ilocos Norte; Dr. Mary Elizabeth Miranda (+639175604744) from Global Alliance for Rabies Control Asia; or Dr. Sergio Recuenco from the Centers for Disease Control and Prevention, United States (1-404-639-0802))with questions or concerns about the study.

Mabalinyo ti tumawag kenni Dr. Loida Valenzuela (+639189337255) iti Opisina ti Beterinaryo Probinsiyal ti Ilocos Norte; Dr. Mary Elizabeth Miranda (+639175604744) iti Global Alliance for Rabies Control Asia; wenno kenni Dr. Sergio Recuenco iti Centers for Disease Control and Prevention, Estados Unidos (1-404-639-0802) maipanggep kadagiti saludsod wenno *concerns* yo maipanggep iti daytoy a panagsaliksik.

**WHAT HAPPENS IF YOU DON’T TAKE PART OR WANT TO STOP?**

**ANA TI MAPASAMAK NO DIKAY MAKIPAGPARTISIPAR WENNO KAYATYO NGA ISARDENG?**

You are free to join the study or not to join.  You may leave the study at any time, for any reason.  Nothing will happen if you decide not to join or to drop out.

Mabalinyo ti libre a makipartisipar iti daytoy a panagsaliksik wenno saan. Mabalinyo met ti agsardeng iti aniaman nga oras, iti uray ana a rason. Awan ti mapasamak kanyayo no saan kayo makipartisipar wenno agsardeng kayo.

**AGREEMENT**

**KATULAGAN**

This study has been explained to me. I have had a chance to ask questions. Any questions I had were answered. I can choose to be in this study. I can drop out of the study at any time. I will receive a copy of this form.
Daytoy a panagsaliksik ket nailawlawag kanyak. Naaddaanak iti gundaway nga agsaludsod. Aniaman a sinaludsodko ket nasungbatan. Mabalinko a pilyen ti mairaman iti daytoy a panagsaliksik. Mabalinko met ti agsardeng iti aniaman nga oras. Makaawatakto iti kopya daytoy a porma.

I am 21 years of age or older and I agree to join the study,

Agtawenak iti 21 wenno nangatngato ket siaannugotak a makipartisipar iti daytoy a panagsaliksik,

**Initial of the interviewee: ___________________ Date:_______________________**

**Inisyal ti nagsaludsodan: Petsa**

**Witness to the initials:______________________ Date:_______________________**

**Paneknekan ni: Petsa**

**_________________________________________ Date:_______________________**

**Printed Name and Signature of Investigator Petsa**

**Perma iti Ngatuen ti Nagan ti Nagsaludsod**

(*space for thumbprint if needed*)

(para iti marka ti tammudo no kasapulan)

(If participant is illiterate, you will need thumbprint and signature of witness)

(No ti pagsaludsodan ket saan a nakapagadal, masapulna ti agdeppel ket agperma met ti mangpaneknek)

**Witness to the initials:______________________ Date:_______________________**

**Paneknekan ni: Petsa**

**_________________________________________ Date:_______________________**

**Printed Name and Signature of Investigator Petsa**

**Perma iti Ngatuen ti Nagan ti Nagsaludsod**
